# Polymorphisms in early neurodevelopmental genes affect natural variation in alcohol sensitivity in adult drosophila

**DOI:** 10.1186/s12864-015-2064-5

**Published:** 2015-10-26

**Authors:** Tatiana V. Morozova, Wen Huang, Victoria A. Pray, Thomas Whitham, Robert R. H. Anholt, Trudy F. C. Mackay

**Affiliations:** Department of Biological Sciences, W. M. Keck Center for Behavioral Biology and Program in Genetics, North Carolina State University, Box 7614, Raleigh, NC 27695 USA; Department of Biochemistry and Physiology, School of Bioscience and Medicine, Faculty of Health and Medical Sciences, University of Surrey, Guildford, Surrey, GU2 7XH UK

**Keywords:** *Drosophila* Genetic Reference Panel, Genome-wide association analysis, Extreme QTL mapping, Alcohol tolerance, Genetic networks

## Abstract

**Background:**

Alcohol abuse and alcoholism are significant public health problems, but the genetic basis for individual variation in alcohol sensitivity remains poorly understood. *Drosophila melanogaster* presents a powerful model system for dissecting the genetic underpinnings that determine individual variation in alcohol-related phenotypes. We performed genome wide association analyses for alcohol sensitivity using the sequenced, inbred lines of the *D. melanogaster* Genetic Reference Panel (DGRP) together with extreme QTL mapping in an advanced intercross population derived from sensitive and resistant DGRP lines.

**Results:**

The DGRP harbors substantial genetic variation for alcohol sensitivity and tolerance. We identified 247 candidate genes affecting alcohol sensitivity in the DGRP or the DGRP-derived advanced intercross population, some of which met a Bonferroni-corrected significance threshold, while others occurred among the top candidate genes associated with variation in alcohol sensitivity in multiple analyses. Among these were candidate genes associated with development and function of the nervous system, including several genes in the *Dopamine decarboxylase* (*Ddc*) cluster involved in catecholamine synthesis. We found that 58 of these genes formed a genetic interaction network. We verified candidate genes using mutational analysis, targeted gene disruption through RNAi knock-down and transcriptional profiling. Two-thirds of the candidate genes have been implicated in previous Drosophila, mouse and human studies of alcohol-related phenotypes.

**Conclusions:**

Individual variation in alcohol sensitivity in Drosophila is highly polygenic and in part determined by variation in evolutionarily conserved signaling pathways that are associated with catecholamine neurotransmitter biosynthesis and early development of the nervous system.

**Electronic supplementary material:**

The online version of this article (doi:10.1186/s12864-015-2064-5) contains supplementary material, which is available to authorized users.

## Background

As a common by-product of natural fermentation, alcohol has been an integral part of human culture since early recorded history. The inebriating effects of alcohol have been recorded since biblical times. In today’s society, excessive alcohol consumption is the most widespread substance abuse problem with substantial socioeconomic impact.

Different human populations vary in alcohol consumption and in susceptibility to the physiological effects of alcohol, as do individuals within populations [1–4]. The extent to which individuals develop tolerance to the physiological effects of alcohol is a contributing factor to the development of addiction. Studies on genetic susceptibility to the physiological effects of alcohol in human populations have mostly focused on aspects of addiction. Linkage and association studies for candidate genes [5–10] as well as genome-wide analyses [10–15] have implicated neurotransmitter systems associated with the mesolimbic reward pathway and enzymes that contribute to alcohol metabolism. However, results from genome-wide association (GWA) studies in people, as well as information from studies on animal models, indicate that additional regulatory processes are involved in alcohol addiction and that the genetic architecture that predisposes individuals to addiction is complex [5, 16–20].

Although addiction is undeniably an important alcohol-related phenotype, acute intoxication is a major contributor to the socioeconomic costs of alcohol abuse. Few studies have focused on acute alcohol sensitivity within the range of normal alcohol intake across a population. Disentangling the genetic and environmental contributions that shape alcohol-related phenotypes in human populations is challenging because alcohol exposure and other contributing environmental factors cannot be controlled. Further complications arise because different studies have utilized different measurements to document alcohol-related phenotypes. Partitioning the relative contributions of genotype, ethanol exposure and the interaction between genotype and environment is more readily accomplished in animal models in which both the genetic background and environment can be controlled and alcohol-related phenotypes quantified accurately. Based on the principle of evolutionary conservation, animal models can provide general insights in fundamental biological processes affected by alcohol exposure [16, 18, 21–24].

*Drosophila melanogaster* provides such a model. Assays have been developed to precisely quantify sensitivity to alcohol by measuring alcohol-induced knock-down time [25, 26], and flies exposed to ethanol undergo physiological and behavioral changes that resemble human alcohol intoxication [27, 28]. Previous studies on alcohol sensitivity in Drosophila have identified cyclic AMP signaling [29, 30], alcohol metabolism (ADH) [31–33] and malic enzyme activity [34, 35] as focal pathways that mediate responses to alcohol exposure. Malic enzyme serves as a metabolic switch to redirect energy metabolism toward fatty acid biosynthesis. Results from the Drosophila model guided the identification of association between polymorphisms in cytosolic malic enzyme with human alcohol consumption in the Framingham Heart Study Offspring cohort [34]. The genetic architecture that underlies alcohol sensitivity and induction of tolerance, however, is more complex; it involves transcriptional regulators that alter gene expression [35–37] and consists of intricate genetic networks [34, 38]. In a previous study we have shown that acute exposure to ethanol results in altered transcript abundance levels of chemoreceptor and detoxification genes, whereas the subsequent development of tolerance is accompanied by changes in transcript abundances of metabolic enzymes [35]. However, most studies on the genetic basis of alcohol sensitivity have focused on effects of single genes. A major challenge in understanding the biological effects of alcohol is to identify the interacting networks of segregating loci that contribute to natural variation in alcohol sensitivity.

Here, we performed two complementary genome-wide association analyses to explore the genetic basis of natural variation in alcohol sensitivity in Drosophila. We performed genome wide association (GWA) analyses for alcohol-related phenotypes using the sequenced inbred lines of the *Drosophila melanogaster* Genetic Reference Panel (DGRP) [39, 40], and extreme QTL mapping analyses [41, 42] utilizing an advanced intercross population (AIP) derived from sensitive and resistant DGRP lines. We identified 247 high confidence candidate genes and placed them in genetic and physical interaction networks. These candidate genes are associated with development and function of the nervous system and include several genes involved in catecholamine synthesis. We confirmed the role of several candidate genes in alcohol sensitivity by functional analyses of mutations and gene expression levels. Remarkably, 66.8 % of the candidate genes have been implicated in previous Drosophila, mouse and human studies of alcohol-related phenotypes, suggesting that these candidate genes and genetic networks are associated with evolutionarily conserved processes that underlie natural variation in sensitivity to alcohol.

## Results

### Natural variation for alcohol sensitivity

**Fig. 1:**
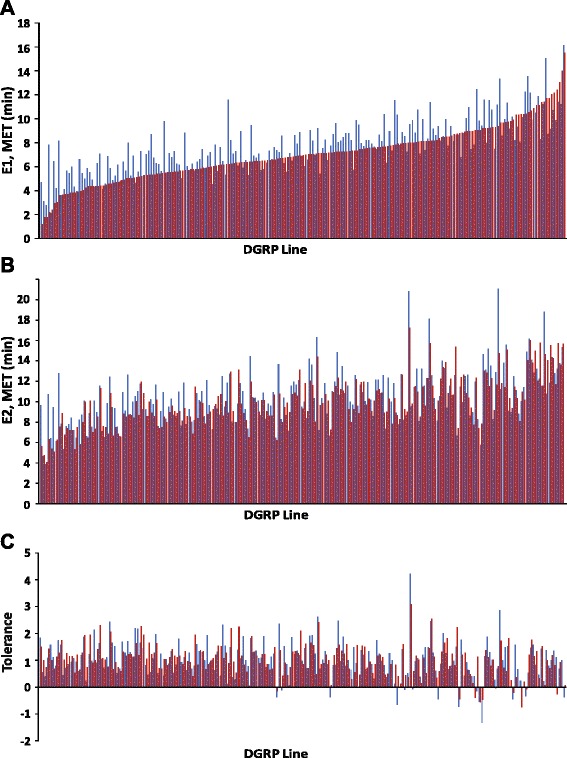
Variation in Mean Elution Time (MET) among the 205 inbred lines of the *Drosophila melanogaster* Genetic Reference Panel (DGRP). **a** Variation in response to initial alcohol exposure (E1). **b** Variation in response to a second alcohol exposure (E2). **c** Variation in tolerance. Red bars represent females and blue bars males

We took advantage of natural variation in the DGRP to analyze phenotypic variation in responses to acute and repeated exposures to ethanol. We measured alcohol knockdown time (Mean Elution Time, MET) in an “inebriometer” [34, 35] after a single (acute) alcohol exposure (E1), and after a second exposure (E2) following a 2 h recovery period, separately for males and females. We found considerable genetic variation in alcohol sensitivity among the DGRP lines (Fig. 1, Additional file 1: Table S1, Additional file 2).

We expressed individual METs of the 205 DGRP lines as deviations from the control mean of the *Canton S* (B) line for the appropriate date and sex. Over the course of the experiments, the *Canton S* (B) elution time after a single ethanol exposure has a mean of 6.1 ± 0.1 and 6.05 ± 0.1 min for males and females, respectively. The METs after the first exposure ranged from 1.2 to 15.5 min in females and 2.1 to 16.1 min in males (*P*_*Line*_ < 0.0001), with a broad sense heritability of *H*^*2*^ = 0.42. The averages for DGRP males and females are 7.75 ± 0.02 and 7.05 ± 0.02 min, respectively. The *Canton S* (B) males and females ranked 69/(205 + 1) and 49/(205 + 1) among the DGRP lines.

The METs after the second exposure were on average higher, reflecting the development of tolerance, and ranged from 4.0 to 17.4 min in females and 3.9 to 21 min in males (*P*_*Line*_ < 0.0001), with *H*^*2*^ = 0.38 (Fig. 1, Additional file 1: Table S1). Genetic variation for alcohol sensitivity is partially context-dependent. Although the genetic (Additional file 1: Table S1) and phenotypic (Additional file 3: Figure S1) correlations are high between males and females for E1 and E2, and between E1 and E2 for males and females, the significant Line × Sex and Line × Exposure interaction terms (*P* < 0.0001) (Additional file 1: Table S1) reflect genetic variation in the magnitude of sexual dimorphism in E1 and E2 as well as genetic variation in the magnitude of induction of tolerance in the two sexes. Genetic variation in the induction of tolerance can be quantified for each line as the scaled difference in mean elution time between the second and first exposures [43].

Induction of tolerance ranged from −0.75 to 4 min and −1.3 to 4 min for females and males, respectively. Several lines were unable to develop tolerance, and, among the majority that did, the magnitude of tolerance varied continuously (Fig. 1).

### GWA analyses for alcohol sensitivity in the DGRP

We performed genome-wide single variant association tests for sensitivity to acute and repeated ethanol exposures as well as induction of tolerance for the DGRP lines, using 1,891,456 DNA sequence variants with minor allele frequencies greater than 0.05 [39]. We performed these analyses for males and females separately within each treatment, as well as using the differences in alcohol sensitivity between the sexes as phenotypic values to identify alleles that modulate sexual dimorphism in alcohol sensitivity. We considered variants with a *P-*value smaller than 5 × 10^−5^ to be nominally significant (Additional files 4, 5 and 6).

Given the large numbers of tests performed and the relatively small number of DGRP lines, only variants with large effects can achieve significance following a strict Bonferroni correction for multiple tests (*P* < 2.64 × 10^−8^). Three closely linked SNPs in strong linkage disequilibrium (LD) between *3L*_2180717 and *3L*_2180921, in a gene desert approximately 8 kb upstream of *CG15820* and 5 kb downstream of *CG13810*, affect female sensitivity to an acute alcohol exposure; and one SNP at *X*_7651622 in the second intron of *ct* affects male sensitivity to a second exposure at the Bonferroni-corrected significance threshold. *ct*, an early developmental homeobox transcription factor with pleiotropic effects on multiple phenotypes, has been implicated in alcohol sensitivity in Drosophila [44], as have its orthologs in mice [45, 46] and humans [14, 47].

The genetic correlations of METs between ethanol exposures and males and females are high. Therefore, the different analyses partially serve as independent replicates of alcohol sensitivity, and variants and genes in common among the top ranking associations from the different analyses are likely to be true positives, even though they do not achieve formal statistical significance in their individual analyses. Indeed, 75 genes were tagged by top variants in one or more treatment/sex combinations (*P* = 0.001 by 1,000 permutations; Additional file 7). Remarkably, 57 (70 %) of these genes were previously associated with alcohol sensitivity in Drosophila, mice and/or humans, and five have been functionally validated previously in Drosophila (Additional file 7).

### Extreme QTL Mapping

The advantage of the Drosophila system is that we can construct a trait-specific, advanced intercross outbred population (AIP) from extreme DGRP lines. The sample size of the AIP is not limiting, giving increased power to detect variants with smaller effects than in the DGRP. Further, the DGRP GWA analyses focused on common variants, because rare alleles are prone to false positives [39], but may give rise to large phenotypic effects [48]. In contrast, any variant private to one of the parental lines used to construct the AIP will be at intermediate frequency if the number of parental lines is small, thus enabling evaluation of the contribution of alleles with MAF < 0.05 to natural variation in alcohol sensitivity. Finally, the AIP serves as an independent validation of candidate genes identified in the DGRP.

**Fig. 2:**
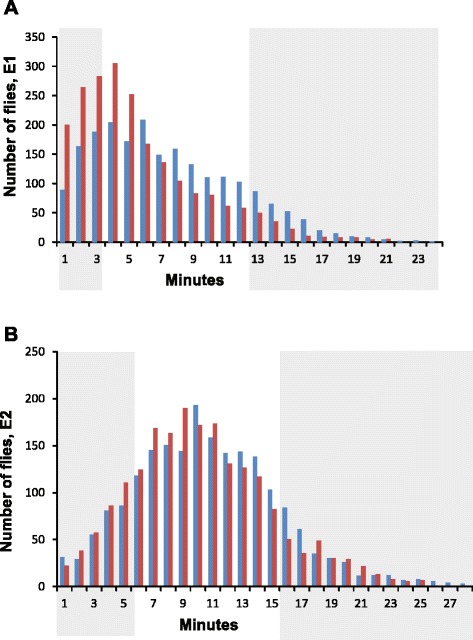
Extreme QTL mapping analysis. Distribution of elution profiles for the advanced intercross population after an acute ethanol exposure (**a**) and after two ethanol exposures separated by a 2 h interval (**b**). Extremes (10 %) of the distributions were used for extreme QTL mapping and are shown by the shaded areas. Blue bars indicate males, red bars indicate females

We constructed an AIP for alcohol sensitivity by crossing six DGRP lines with divergent and extreme alcohol sensitivities (three sensitive and three resistant lines for both E1 and E2) to generate a base population in which all lines were equally represented. We maintained this population by random mating at large census size for over 25 generations. Beginning at generation 25, we scored 2,000 males and 2,000 females from the AIP for ethanol sensitivity after acute (E1) or repeated (E2) ethanol exposures, and selected the 10 % most sensitive and resistant males and females for each treatment (Fig. 2). We performed bulk DNA sequencing on DNA pooled from these extreme individuals and performed an extreme QTL mapping GWA analysis [41, 49] by comparing differences in allele frequencies between the pools. Alleles with divergent frequencies between sensitive and resistant DNA pools are either themselves causal alleles or they are in LD with causal alleles affecting the trait.

Consistent with the increased power of extreme QTL mapping in an AIP, we found a total of 60 variants in or near 53 genes associated with alcohol sensitivity in the four extreme QTL GWA analyses (Additional files 7 and 8) at a Bonferroni-corrected significance threshold (*P* < 5 × 10^−8^) with a total of 1,461 variants passing a nominal threshold of *P* < 10^−5^. Notably, several of these genes have been implicated previously in Drosophila alcohol-related phenotypes, including *bun*, *Catsup*, *CG2064*, *CG2065*, *CG3326*, *CG9005*, *CG9674*, *for*, *ham*, *hig*, *Lar*, *Lim3*, *nudC*, *Pde11*, *toc* and *vir-1* [34–38, 44, 50, 51] (Additional file 7). Several of these are located in the *Ddc* gene cluster on chromosome *2 L*, which harbors closely linked genes involved in catecholamine metabolism.

The *Ddc* gene cluster is of particular interest with regard to alcohol sensitivity in Drosophila. Dopamine has been implicated in aversive conditioning towards repellent odorants [52, 53]. However, dopaminergic neurotransmission appears to be essential for development of conditioned preference for ethanol-associated cues [54]. Both octopamine and dopamine have been implicated in appetitive reward signaling in response to a sugar stimulus [55, 56]. Octopamine-mediated reinforcement depends on interactions with dopaminergic neurons in the mushroom bodies [55, 56]. Dopaminergic neurons in the protocerebral anterior medial cluster that project to the medial lobes of the mushroom bodies [55] have been implicated in appetitive reinforcement. Thus, this dopamine-mediated positive reinforcement circuit may represent an insect analog of the vertebrate mesolimbic dopamine reward system that originates in the nucleus accumbens.

### Comparison of DGRP and extreme QTL GWA analyses

Applying the same logic to the extreme QTL GWA analysis as for the DGRP GWA analyses, the top candidate genes that do not meet the Bonferroni significance threshold will occur in more than one analysis. We found 119 genes tagged by top variants in one or more treatment/sex combinations in the extreme QTL GWA analysis (*P* = 0.001 by 1,000 permutations; Additional file 7), of which 84 (70 %) were previously associated with alcohol sensitivity in Drosophila, mice and/or humans, and four have been functionally validated previously in Drosophila (Additional file 7).

No polymorphisms were in common between the top associations in the DGRP GWA analyses and the extreme QTL GWA analyses. This is not unexpected, since many of the polymorphisms associated with alcohol sensitivity phenotypes in the DGRP are not present in the six founding parents of the AIP, and low frequency variants that could not be tested in the DGRP that are present in the founding parents have a frequency of at least 0.167 in the AIP, and can be tested. In addition, we induced LD by crossing the six parental lines in the first generation of the AIP, and thus the local LD structure differs between the DGRP and AIP. However, even if no polymorphisms are in common, under an additive model we do expect overlap among the top genes detected in the two GWA analyses. Indeed, we found 62 genes in common between the DGRP and extreme QTL analyses, of which 42 were previously associated with alcohol sensitivity in Drosophila, mice and/or humans, and four have been functionally validated previously in Drosophila (Table 1; Additional file 7).

**Table 1:** Common candidate genes for DGRP and extreme QTL GWA analyses previously associated with alcohol-related phenotypes in humans

Drosophila gene symbol	Biological process	Human gene symbol
*CAP*	Muscle attachment; sensory perception of sound	*CAP2* [82]
*CG31690*	-	*TMTC2* [63]
*Dys*	Imaginal disc-derived wing vein morphogenesis; neuromuscular synaptic transmission; muscle organ development	*DMD, UTRN* [14, 63]
*fz*	Wnt signaling pathway; signal transduction; axon extension; heart development; negative regulation of Notch signaling pathway; establishment or maintenance of cell polarity	*FZD9* [82]
*IA-2*	Protein dephosphorylation; regulation of secretion	*PTPRN2* [14]
*KCNQ*	Potassium ion transport; embryonic development via the syncytial blastoderm; regulation of heart rate	*KCNQ3* [63, 64]
*luna*	Regulation of transcription, DNA-templated; mitotic sister chromatid segregation	*KLF7* [110]
*milt*	Axon transport of mitochondrion	*TRAK2* [63]
*mtt*	Phospholipase C-activating G-protein coupled receptor signaling pathway; response to insecticide; adult feeding behavior	*GRM5* [12]
*Nrx-IV*	Dorsal closure; protein localization; synaptic vesicle targeting; cell-cell junction organization; establishment or maintenance of cell polarity	*CHRNA5, CHRNA 7* [10, 83, 111]
*Pde1c*	cGMP metabolic process; cAMP metabolic process	*PDE1C* [63, 64]
*Rbp6*	Stem cell development	*MSI2* [112]
*rhea*	Cell adhesion; muscle attachment; regulation of cell shape; larval somatic muscle development; negative regulation of transcription, DNA-templated; phagocytosis	*TLN2* [63]
*shn*	Ectoderm development; cell proliferation; learning or memory; olfactory learning; transforming growth factor beta receptor signaling pathway; peripheral nervous system development; positive regulation of transcription from RNA polymerase II promoter	*HIVEP1* [63]
*stan*	Establishment of planar polarity; cell adhesion; axonogenesis; mushroom body development; Wnt signaling pathway; negative regulation of Notch signaling pathway	*CELSR1* [113]

### Interaction networks for alcohol sensitivity

**Fig. 3:**
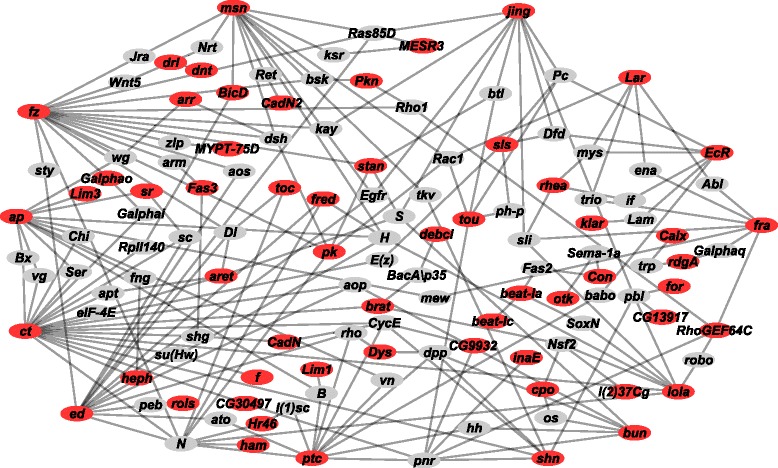
Genetic interaction network for variation in alcohol sensitivity. The network was constructed from candidate genes (red ovals), identified from the combined DGRP and extreme QTL GWA analyses, while allowing for one missing gene (grey ovals). The network consists of 58 interconnected candidate genes

In total, we identified 247 candidate genes for ethanol sensitivity as those genes with at least one variant significant at a Bonferroni-corrected *P*-value, as well as genes among the top associations that occurred in more than one of the DGRP, extreme QTL, or both the DGRP and extreme QTL GWA analyses (Additional file 7). The top candidate genes are highly pleiotropic and for many, genetic and physical interaction partners are known [57, 58] (Additional files 9 and 10). We used this information to place the candidate genes in context. A total of 58 candidate genes participate in a genetic interaction network of 127 genes (allowing one missing gene not implicated by our GWA analyses) (Fig. 3). This network includes elements of signaling pathways associated with early development, including canonical Notch, Wingless, Epidermal Growth Factor, and Hedgehog signaling pathways. Genes involved in cyclic nucleotide signaling, which has been previously associated with alcohol sensitivity [29, 59–62], are also present in this network. Many of the candidate genes interact with each other and may be control points that participate in a large number of additional interactions. For example, *ct*, which was tagged by a SNP that achieved Bonferroni-level significance in the DGRP GWA analysis, interacts with *lola*, which was implicated in the extreme QTL mapping GWA analysis. *ct* and *lola* both encode transcription factors; *ct* has been implicated as a target for Notch signaling in wing development, and *lola* is a major regulator of axon guidance. Similarly, a total of 95 candidate genes participate in a physical interaction network of 617 genes (again allowing for one missing gene) (Additional file 11: Figure S2).

### Functional validation of candidate genes

**Fig. 4:**
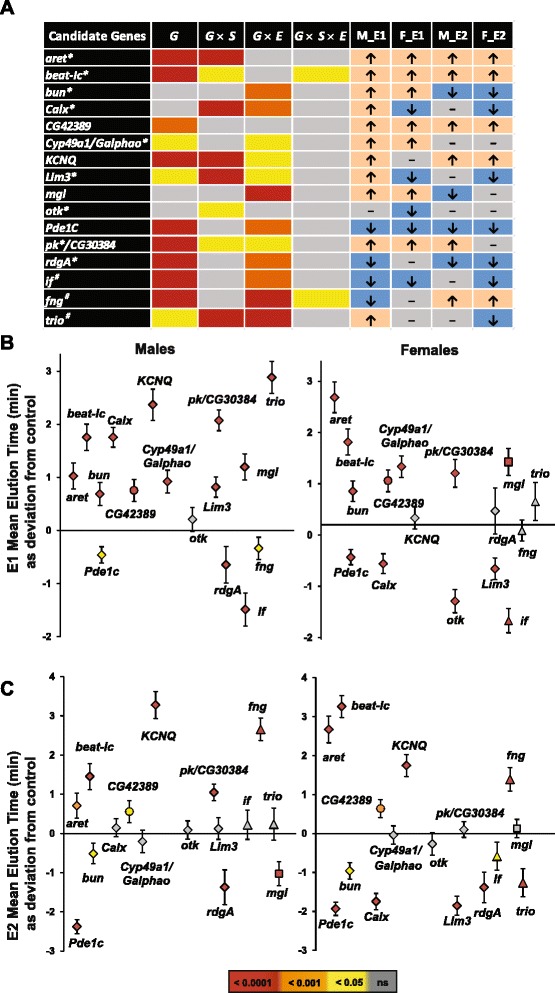
Functional confirmations of candidate genes. **a** Candidate genes from the DGRP, extreme QTL GWA analyses and the genetic network analysis were functionally tested using transposon insertion mutations and RNAi knockdown lines. All 16 candidate genes showed significant differences from the control for at least one of the genetic terms (*G*, *G* × *S*, *G* × *E*, *G* × *S* × *E*). *G* – Genotype: mutant or control; *S* – Sex: Male, Female; *E* − Exposure: E1, E2; * - genes present in genetic network from Fig. 3; *#* – missing gene from the genetic network. M_E1, M_E2, F_E1 and F_E2 – phenotypic effects in males (M) and females (F), respectively after an acute (E1) or repeated (E2) ethanol exposures. **b** Effects of 16 mutants of candidate genes on sensitivity to acute (top panel) and repeated (bottom panel) ethanol exposure in males (left) and females (right) compared to control. Squares indicate candidate genes found only in GWA analyses; circles indicate candidate genes found only from extreme QTL mapping analyses; Triangles indicate missing genes from the genetic network in Fig. 3. Candidate genes found in more than one analyses (GWA, extreme QTL mapping and network analysis) are shown with diamond shapes. Data are presented as the deviation of the MET of each line and sex from the appropriate control line ± SEM, calculated as $\sqrt{SE{m}^2+SE{c}^2}$, where *m* and *c* are SE for mutant and control lines, respectively. The color bar indicates the significance levels for both Fig. 4a and b

We used mutational analysis to establish causal links between genes implicated in our DGRP and extreme QTL GWA analyses and effects on alcohol sensitivity. Based on availability of co-isogenic *Mi{ET1}* and *P*-element insertional mutations, we tested mutations in *aret*, *beat-Ic*, *bun*, *Calx*, *CG42389*, *if*, *Cyp49a1/Galphao*, *Lim3*, *mgl*, *otk*, *Pde1C*, *pk/CG30384*, *rdgA* and *trio*. In addition, we tested *UAS-RNAi* lines under an *Ubi-GAL4* driver targeting *KCNQ* and *fng*. We found that *aret*, *beat-Ic, CG42389, Cyp49a1/Galphao, KCNQ* and *pk/CG30384* showed increased resistance after acute and/or repeated ethanol exposures; *Pde1C, if, otk and rdgA* were more sensitive to ethanol exposures; *bun, Calx, fng, Lim3, mgl* and *trio* demonstrated either increased or decreased resistance to ethanol depending on the sex or number of ethanol exposures (Fig. 4a–b). In summary, all 13 candidate genes and the three missing genes implicated by the network analysis (*if*, *fng* and *trio*) had significant effects on ethanol sensitivity in at least one sex/exposure combination, indicating that our top candidate genes are enriched for true positive associations. Orthologs of eight of these functionally validated genes (*bun*, *Calx*, *Galphao*, *KCNQ*, *Lim3*, *mgl*, *Pde1C, rdgA*) have previously been associated with alcohol phenotypes in mouse and human studies (Additional file 7, [45, 46, 63–66]), suggesting that inferences made from Drosophila are more broadly relevant across taxa.

**Fig. 5:**
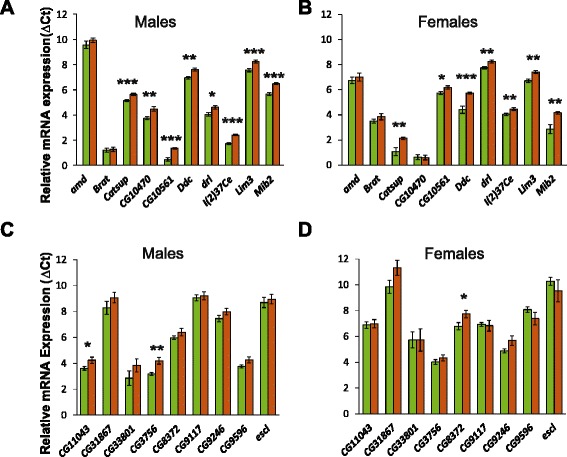
Expression levels of mRNAs of a group of genes located on chromosome *2 L* pooled across parental lines (three sensitive *vs* three resistant lines) used to establish the advanced intercross population. Panels (**a**) and (**b**) show mRNA expression levels of candidate genes from extreme QTL mapping analyses in males and females, respectively. Panels (**c**) and (**d**) show mRNA expression levels of randomly selected genes in males and females, respectively. Green bars indicate the pool of sensitive lines, orange bars indicate the pool of resistant lines. *: *P* < 0.05; **: *P* < 0.001; ***: *P* < 0.0001; Student’s *t*-test

The extreme QTL GWA analysis identified a cluster of 21 genes located on chromosome *2 L* between two intergenic regions (*2 L*: 19,034,444 - 19,208,621) spanning from *mib2* to *drl* and associated with variation in acute alcohol sensitivity (Additional file 8), including *Catsup* and *Ddc*, which regulate the biosynthesis of dopamine, a neurotransmitter critically associated with alcohol addiction in human studies [67, 68] and implicated in associative conditioning in Drosophila [52, 53]. We focused only on candidate genes that have SNPs in introns, coding or 3’ and 5’ UTR regions. We analyzed expression levels of ten candidate genes (*amd*, *brat*, *Catsup*, *CG10470*, *CG10561*, *l(2)37Ce*, *Ddc*, *drl*, *mib2* and *Lim3)* in pools of the three extreme sensitive and three extreme resistant DGRP lines that were used to generate the advanced intercross population by quantitative RT-PCR for both sexes, separately. We found that 15 of 20 (75 %) transcripts tested indeed showed altered transcript levels between the sensitive and resistant pools for both sexes (Fig. 5a–b). It is of interest to note that increased expression of the genes indicated in Fig. 5 (a–b) is associated with increased resistance. To assess whether this is significantly different from random expectation, we also measured transcript abundance levels of nine randomly selected genes that are also located on chromosome *2 L* and have not been associated with alcohol sensitivity previously. Here, only 3 of 18 (17 %) transcripts showed altered transcripts level between the extreme pools (Fig. 5c–d). The proportion of candidate genes with altered expression level is significantly greater than for randomly selected genes (Fisher’s exact test, *P* = 0.039). Thus, differences in expression levels of these candidate genes could be a causal explanation for their association with variation in acute alcohol sensitivity.

In summary, our functional analyses suggest that networks of early neurodevelopmental genes as well as variation in catecholamine neurotransmitter levels determine natural variation in alcohol sensitivity in adult flies in this wild derived population.

## Discussion

Alcohol-related phenotypes are diverse and span the spectrum from acute intoxication to long-term physiological addiction. Similarly, the physiological effects of alcohol are diverse, encompassing both intermediary metabolic pathways as well as the nervous system, where alcohol can exert both general sedative effects and elicit addiction through its actions on the dopaminergic reward system. The complexity of the physiological responses to alcohol exposure *a priori* predict that multiple interacting genetic factors would predispose to alcohol sensitivity; thus, a comprehensive understanding of the genetic underpinnings of alcohol sensitivity must reach beyond studies of individual genes, but take into account a genetic context in which environmentally sensitive ensembles of genes determine the phenotypic outcome.

Studies on alcohol drinking behavior, intoxication, and addiction in human populations or in mouse models have focused primarily on QTL mapping [69–72], candidate gene associations [73–81], transcriptional profiling [45–47, 65, 82–84] and, most recently, on genome-wide associations [10, 12, 14, 20, 63, 85–87]. Previous studies on the genetics of alcohol sensitivity in Drosophila employed mutagenesis screens [38], transcriptional profiling of flies before and after alcohol exposure [35, 36] or of lines artificially selected for high and low alcohol sensitivity [37], as well as characterization of selected candidate genes [30, 33, 51, 88–96]. Here, we have used the DGRP and a DGRP-derived AIP to perform the first GWA analyses to identify genes harboring variants that determine natural variation in individual alcohol sensitivity and variation in induction of tolerance in the Drosophila model.

Combining the results from both GWA analyses identified candidate genes associated with natural variation in alcohol sensitivity with a high degree of confidence. Causal relationships of a large fraction of these genes with phenotypic variation is apparent from the high degree with which mutations in those candidate genes recapitulate effects on alcohol related phenotypes and from previous studies which have implicated the same genes in alcohol related phenotypes both in Drosophila and vertebrates, including mice and humans (Additional file 7). Most of the genes involved in cell adhesion, neural development, cyclic nucleotide and inositol triphosphate metabolism and signaling emerged as candidate genes from prior studies as well. Human orthologs of *for*, *KCNQ*, *Pde1C*, *phl*, *Pkc53E*, *rhea* [58], *fra* [63], *Nrx-IV* [10], *Pde11* [59], *Pka-R2* [60] and *RhoGAP68F* [14] have been associated with alcohol dependence and/or alcohol consumption in human GWA studies.

We were able to connect candidate genes associated with variation in alcohol sensitivity in a genetic interaction network that encompasses early neurodevelopmental signaling pathways, including Notch, Wnt, EGFR, and Hedgehog pathways (Fig. 3). It is of interest to note that *ct*, which is a target of the Notch pathway [97, 98] and implicated in axonogenesis [99, 100], shows an association that surpasses the stringent Bonferroni-corrected multiple testing threshold in the GWA analysis of the DGRP. Thus, subtle differences in neuronal connectivity determined in early development of the nervous system or possibly reflecting variation in neuroplasticity in the adult brain appear to be a major determinant of individual variation in susceptibility to alcohol exposure. Together with the genetic network that regulates neurodevelopment, our analyses implicate genes associated with neurotransmitter signaling, including dopamine and the cyclic AMP signaling pathway as factors that affect individual variation in alcohol sensitivity.

Three important insights emerge from our analyses. First, the majority of previous studies have identified widespread changes in genome wide transcript expression on exposure to alcohol [34–38]. However, due to the highly correlated nature of transcriptional co-regulation networks, it is not possible to infer which changes are causal and which are co-regulated responses to causal changes. Comparison of our results with these studies (Additional file 7) indicates that the genes we identify as top candidates based on genetic polymorphisms in the GWA analyses largely overlap with genes whose expression is up- or down-regulated in response to alcohol, suggesting that these genes may be causal, affecting both transcriptional responses and whole organism behavioral responses to alcohol exposure. This hypothesis is amenable to direct experimental testing in the future. Second, the neurodevelopmental genes associated with variation in alcohol sensitivity in the Drosophila system are evolutionarily highly conserved and, thus, it is reasonable to postulate that similar orthologous networks may be associated with variation in alcohol sensitivity across phyla, including in human populations. Further, it is reasonable to surmise that alcohol-induced perturbation of early developmental networks may represent a likely target for the induction of fetal alcohol syndrome in people. Third, principles of pleiotropy and context dependence (including gene-gene interactions, sex-dependence and gene-environment interactions) are generally applicable elements that are likely characteristics of network organization across phyla.

Finally, we should note that we did not detect several genes implicated by artificial selection and mutagenesis screens for alcohol related phenotypes in Drosophila (*e.g. Adh, Aldh* [32, 33], *amnesiac* [29], *white rabbit* [101], *hangover* [102]). There could be several reasons why variants in these genes are not detected in our genome-wide association study. First, functional polymorphisms in these genes may have low frequencies in the population and thus are not captured by our GWA analysis which tests only variants with MAF > 0.05. Second, the effects of the common variants in these genes may be small and our GWA is not sufficiently powered to detect them at the threshold used. Third, the effects of variants in these genes may be buffered by other genes in the complex physiological process of ethanol detoxification. To summarize, we can only uncover genes associated with natural variation in alcohol sensitivity that harbor polymorphisms with allele frequencies and effect sizes large enough to resolve in our analyses. Thus, genes in which mutations clearly have large effects on a trait may not be functionally variable in natural populations if they are under strong selective constraint, or they may harbor rare alleles that cannot be individually assessed by GWA analyses.

## Conclusions

GWA association analyses for ethanol sensitivity/resistance and toerance in the inbred, sequecned DGRP lines and an AIP derived from extremely sensitive and resistant lines identified mutliple genes affecting alcohol-related behaviors. These genes participate in known genetic and physical interaction networks; are in evolutionrily conserved signalling pathways, including catecholamine neurotransmitter biosynthesis and early development of the nervous system; and have been implicated in alcohol-related phenotypes in mice and humans.

## Methods

### Drosophila stocks

The 205 inbred, sequenced lines of the *Drosophila melanogaster* Genetic Reference Panel (DGRP) [39, 40] were derived by 20 generations of full-sib mating from isofemale lines that were collected from the Raleigh, NC farmer’s market. The DGRP contains 4,853,802 single nucleotide polymorphisms (SNPs) and 1,296,080 non-SNP variants (insertions and deletions) as well as 16 large polymorphic chromosomal inversions [39].

*Mi{ET1}* mutants (*aret*^*25234*^, *beat-Ic*^25326^, *Calx*^26124^, *CG42389*^*25307*^, *if*^*29896*^, *Cyp49a1*/*Galphao*^*24593*^, *Lim3*^*23505*^, *mgl*^*26407*^, *otk*^*25334*^, *Pde1C*^*24064*^, *pk/CG30384*^*29252*^, *rdgA*^*26061*^, *trio*^*29073*^) and their co-isogenic control *w*^*1118*^_*iso*_*; 2*_*iso*_*;3*_*iso*_^*5905*^ [103, 104] as well as the *P{GT1}* mutant (*bun*^*12584*^) [105] were obtained from the Bloomington Drosophila Stock Center. The RNAi transgenic fly lines *KCNQ*^*106655*^ and *fng*^*51977*^ as well as the progenitor control lines *y,w*^*1118*^*;P{attP,y*^*+*^*,w*^*3`*^*}*^*60100*^ and *w*^*1118*^_*iso*_*; 2*_*iso*_*;3*_*iso*_^*60000*^ were obtained from the Vienna Drosophila Resource Center (VDRC; [106]. The ubiquitous *Ubi-Gal4*^*32551*^ driver line was obtained from the Bloomington Drosophila Stock Center. We crossed males from the transgenic *UAS*-RNAi line to virgin females from the driver line to suppress the expression of the target gene in hybrid F1 offspring.

Flies were reared under controlled population density and standard culture conditions on cornmeal-molasses-agar medium at 25 °C, 60–75 % relative humidity, 12 h light-dark cycle. Flies were not exposed to CO_2_ anesthesia for at least 24 h prior to assay.

### Quantitative assay for alcohol sensitivity and tolerance

We assessed ethanol sensitivity and tolerance for all 205 DGRP lines. Each day we tested 11 randomly selected DGRP lines and a control line (*Canton S* (B)). There were two replicate measurements for each sex per line with 70 flies per each replicate; the replicates for each line were assessed on different days. To measure alcohol sensitivity we placed 3–5 day old mated males or females in an inebriometer [25] pre-equilibrated with saturated ethanol vapor, and collected them at one-minute intervals as they eluted. We recorded elution times from the initial exposure to ethanol (E1) and two hours later after a second exposure of the same flies (E2). The mean elution time (MET) is a measure of alcohol sensitivity, and the scaled difference of MET between the second and first exposures is a measure of tolerance (T), *i.e.*$T=\left(E{2}_i-E{1}_i\right)/\left(\overset{\_\_\_}{E2}-\overset{\_\_\_}{E1}\right)$, where E2_i_ and E1_i_ are, respectively the MET for E1 and E2 of line *i,* and $\overset{\_\_\_}{E2}$ and $\overset{\_\_\_}{E1}$ are, respectively, the population means of E2 and E1.

### Quantitative genetic and statistical analysis

We expressed individual METs of the 205 DGRP lines as deviations from the control mean of the *Canton S* (B) line for the appropriate date and sex. We used PROC GLM in SAS to partition variance in ethanol sensitivity after the first and second exposures among the inbred lines, pooled across sexes, according to the mixed model *Y* = *μ* + *L* + *S* + *L* × S + *Rep*(*L* × *S*) + *ε*, where *μ* is the overall mean, *S* is the fixed effect of sex, *L* is the random effect of line, *L* × *S* is the random effect of the sex by line interaction, *Rep*(*L* × *S*) is the random effect of replicate, nested within *L* × *S*, and *ε* is the within-replicate (residual) variation. The total genotypic variance among lines was estimated as *σ*_*G*_^2^ = *σ*_*L*_^2^ + *σ*_*L×S*_^2^, where *σ*_*L*_^2^ is the among-line variance component and *σ*_*L×S*_^2^ is the variance attributable to the *L × S* interaction. The total phenotypic variance was estimated as *σ*_*P*_^2^ = *σ*_*G*_^2^ + *σ*_*ε*_^2^, where *σ*_*ε*_^2^ is the environmental variance component. We estimated broad sense heritabilities as *H*^2^ = *σ*_*G*_^2^/*σ*_*P*_^2^. We performed similar analyses separately for each sex, pooled across exposures (*E*): *Y* = *μ* + *L* + *E* + *L* × *E* + *Rep*(*L* × *E*) + *ε*. The total genotypic variance among lines was estimated as *σ*_*G*_^2^ = *σ*_*L*_^2^ + *σ*_*L×E*_^2^, the total phenotypic variance was estimated as *σ*_*P*_^2^ = *σ*_*G*_^2^ + *σ*_*ε*_^2^, and the broad sense heritabilities were estimated as *H*^2^ = *σ*_*G*_^2^/*σ*_*P*_^2^.

### Genome-wide association analyses for alcohol sensitivity and tolerance

We carried out a genome-wide association (GWA) analysis for each of the alcohol sensitivity and tolerance traits using the DGRP web portal (http://dgrp2.gnets.ncsu.edu/; [39]). Briefly, the raw line means were adjusted for effects of *Wolbachia* infection and major inversions, and subsequently used to fit a mixed effects linear model accounting for relatedness among the lines to estimate the effects of individual variants [39]. In total, we tested 1,891,456 variants with minor allele frequencies greater than 0.05.

### Extreme QTL Mapping

To complement the GWA search, we also created an advanced intercross population to perform extreme QTL mapping [49]. To maximize genetic divergence, we selected three lines (DGRP_461, DGRP_721, DGRP_801) with extreme sensitivity and three lines (DGRP_142, DGRP_730, DGRP_908) with extreme resistance to alcohol exposure. Next, we crossed these lines in a partial diallel design to ensure alleles from all lines are equally represented, and maintained this advanced intercross population by random mating with a large effective population size (*N* = 300) for at least 25 generations on standard culture medium. We were able to use the same advanced intercross population for both acute (E1) and repeated alcohol exposure (E2) traits, because of the significant phenotypic correlation between the E1 and E2 phenotypes, with extreme lines being in common between the two traits. In total, we scored 2,000 males and 2,000 females for ethanol sensitivity after acute (E1) or repeated (E2) exposures, beginning at generation 25 and continuing until sufficient sampling was achieved. To measure alcohol sensitivity after the E1 exposure, we placed six sets of 100 3–5 day old mated flies of the same sex in each inebriometer column pre-equilibrated with ethanol vapor, and collected them at one-minute intervals as they eluted. We recorded elution times for each individual fly and selected the 10 % most resistant and the 10 % most sensitive flies. Multiple runs were performed on the same day, with sexes tested on separate days, and the 200 most resistant and most sensitive males and females were collected.

To measure alcohol sensitivity after the E2 exposure we repeated the procedure. Six sets of 100 3–5 day old mated flies of the same sex were placed in the inebriometer, flies were collected as they eluted and the elution times were recorded. Flies were allowed to recover for 2 h, and then re-exposed to ethanol, while recording the elution times and collecting the 10 % extreme flies. We collected the 200 most extreme flies over several days with sexes being scored on separate days.

DNA from the 200 most resistant and most sensitive males and females was extracted from pools of sexes separately using the Genomic-tip 100/G columns (Qiagen). Libraries were constructed from from 250 ng of purified DNA from each of the eight pools (2 exposures x 2 sexes x 2 pools), bar-coded (NEXflex™ ChIP-seq Barcodes, Bioo Scientific), and sequenced on the Illumina HiSeq 2000 platform using 100 bp paired end sequencing in a total of eight lanes. Sequence reads were aligned to the *D. melanogaster* reference genome using Burrows-Wheeler Aligner (BWA, version 0.6.2 [107]). A maximum of five mismatches were allowed and low quality bases at the end were trimmed with the “-q 13” option in BWA. The alignments were locally realigned, marked for PCR duplicates using GATK (version 2.4 [108]) and Picard tools (version 1.89) before base qualities were recalibrated using GATK. Bases passing a series of quality filters [41] were piled up to obtain counts of alleles at SNP sites where the parental lines segregate. Finally, we tested for differences between the sensitive and resistant pools using a *Z* test, where the test statistic was calculated as $Z=\left({p}_S-{p}_R\right)/\sqrt{p_0\left(1-{p}_0\right)\left(2/n+1/{d}_S + {d}_R\right)}$. In this *Z* test, *p*_*S*_ and *p*_*R*_ represent the estimated allele frequencies in the sensitive and resistant pools, respectively; *p*_*0*_ = (*p*_*S*_ + *p*_*R*_)/2 was the allele frequency under the null hypothesis H_0_: *p*_*S*_ = *p*_*R*_; *n* was the total number of chromosomes in each pool; and *d*_*S*_ and *d*_*R*_ were the sequencing depths in the sensitive and resistant pools, respectively. *P-*values were obtained assuming that the *Z* statistic was normally distributed under H_0_. We tested a total of 1,007,811 and 978,002 SNPs in the E1 analyses of females and males, respectively; and 967,331 and 1,007,940 SNPs in the E2 analyses of males and females respectively. We used *P* < 5 × 10^−8^ as an average Bonferroni-corrected *P*-value for all analyses.

### Bioinformatics analysis

We annotated DNA variants using the gene models in Flybase release 5.49 [57]. We mapped genes to the physical and genetic interaction databases downloaded from Flybase. We then extracted subnetworks from the global networks whose edges were either a direct connection between candidate genes or bridged by only one gene not among the candidate gene list.

### Mutant analyses

Mutations in 16 candidate genes were selected for functional assessment. *Minos* and *P-*element insertional mutations as well as a VDRC RNAi line and their co-isogenic controls were measured for alcohol sensitivity after one (E1) and two (E2) exposures, with five replicates (*N* = 80 flies per replicate) per genotype, sex, and exposure. We performed factorial fixed effect ANOVAs of form *Y* = *μ* + *G* + *S* + *E* + *G* × *S* + *G* × *E* + *S* × *E* + *G* × *S* × *E* + *Rep*(*G* × *S × E*) + *ε* to assess the differences between mutant and control genotypes (*G*), males and females (*S*) and alcohol exposures (*E*), where *ε* is the residual variance. Significance of any of the genetic terms (*G*, *G* × *S*, *G* × *E*, *G* × *S* × *E*) indicates an effect of the mutation on MET in at least one condition and shown with the color bar at the bottom of Fig. 4a. Mean elution times for 16 tested candidate genes are presented as the deviation of the MET of each mutant line and sex from the appropriate control line ± SEM, calculated as $\sqrt{SE{m}^2+SE{c}^2}$, where *m* and *c* are SE for mutant and control lines, respectively (Fig. 4b). The color bar indicates the significance level for each test (Student’s *t*-test).

### Assessment of gene expression levels

We quantified mRNA levels by quantitative RT-PCR with the SYBR Green detection method, as described previously [38]. We used *glyceraldehyde-3-phosphate dehydrogenase* as the internal standard. We measured the expression levels of ten candidate genes (*amd*, *brat*, *Catsup*, *CG10470*, *CG10561*, *l(2)37Ce*, *Ddc*, *drl*, *mib2* and *Lim3*) and nine negative control genes (*CG11043*, *CG31867*, *CG33801*, *CG3756*, *CG8372*, *CG9117*, *CG9246*, *CG9596* and *escl*) located on chromosome *2 L*.

Six biological replicates of total RNA was extracted from the same three extreme sensitive (DGRP_461, DGRP_721, DGRP_801) and three extreme resistant (DGRP_142, DGRP_730, DGRP_908) lines used to construct the advanced intercross population, separately for males and females, using the Trizol® Reagent (Ambion). cDNA was generated from 200 ng of total RNA by reverse transcription using the High Capacity cDNA Reverse Transcription Kit with RNase Inhibitor (Applied Biosystems). Primer Express 3 software (Applied Biosystems) was used to design transcript-specific primers to amplify up to 100-bp regions of all genes of interest. Primers were designed to encompass common regions of alternative transcripts, were evaluated for hairpins and polymorphisms in the sequences that potentially may lower the affinity of the primer for the gene sequence. We encounted such problems for primers for *Rpn3* and *CG17572* and excluded them from further analysis (Additional file 12). Negative controls without reverse transcriptase were used to exclude potential genomic DNA contamination. Three technical replicates with six biological replicates across all lines were run on the same 384-well microtiter plate, including *Gpdh* as internal standard. Expression of each gene in each biological replicate for each DGRP line and for each sex was normalized relative to the appropriate *Gpdh* expression level using ∆Ct values, according to ABI User Bulletin no. 2 [109]. Statistically significant differences in gene expression levels between sensitive and resistant pools were determined by Student’s *t*-tests on ∆Ct values.

## Additional files

Additional file 1: Table S1. Analyses of variance of alcohol sensitivity of 205 DGRP lines. (DOCX 20 kb) Additional file 2:
**METs for each DGRP line for males and females, by treatment (E1, E2, T).** (XLSX 73 kb) Additional file 3: Figure S1. Phenotypic correlations among METs in males and females and across treatments. (**A**) E1 females and males (*r*_*P*_ = 0.78). (**B**) E2 females and males (*r*_*P*_ = 0.79). (**C**) Females, E1 and E2 (*r*_*P*_ = 0.7). **D**) Males, E1 and E2 (*r*_*P*_ = 0.7). (**E**) Tolerance, females and males (*r*_*P*_ = 0.67). (PDF 313 kb) Additional file 4:
**GWA analyses of MET in the DGRP following a single ethanol exposure (E1).** All variants significant at a nominal *P*-value < 5 × 10^-5^ in any test are listed. Effects are one-half the mean difference in MET between the major and minor allele classes. (XLSX 69 kb) Additional file 5:
**GWA analyses of MET in the DGRP following a second ethanol exposure (E2).** All variants significant at a nominal *P*-value < 5 × 10^-5^ in any test are listed. Effects are one-half the mean difference in MET between the major and minor allele classes. (XLSX 68 kb) Additional file 6:
**GWA analyses of tolerance (T) in the DGRP.** All variants significant at a nominal *P*-value < 5 × 10^-5^ in any test are listed. Effects are one-half the mean difference in MET between the major and minor allele classes. (XLSX 74 kb) Additional file 7:
**Candidate genes associated with alcohol sensitivity from GWA studies on the DGRP (DGRP_) and/or from the extreme QTL (xQTL_) mapping analyses. F: females, M: males; D: difference between males and females.** E1: acute ethanol exposure; E2: repeated ethanol exposure; T: induction of tolerance. Numbers in parenthesis indicate are the number of shared polymorphism in the same gene. References to studies in which the same gene or orthologous gene have been implicated in alcohol sensitivity are given in square brackets. (XLSX 37 kb) Additional file 8:
**Extreme QTL mapping GWA analyses in the AIP.** Information is given for the top variants in each analysis, as well as corresponding information for the same variants in the other analyses. *f* denotes allele frequency and subscripts R and S denote resistant and sensitive pools, respectively. E1 is for acute ethanol exposure and E2 indicates the second ethanol exposure. (XLSX 682 kb) Additional file 9:
**Genetic interactions among top candidate genes for alcohol sensitivity.** The candidate gene column indicates which of the interacting partners are candidate genes (Yes) and which have been recruited to the network via their physical interactions with a candidate gene (No). (XLSX 19 kb) Additional file 10:
**Physical interactions among top candidate genes for alcohol sensitivity.** The candidate gene column indicates which of the interacting partners are candidate genes (Yes) and which have been recruited to the network via their physical interactions with a candidate gene (No). (XLSX 78 kb) Additional file 11: Figure S2. Protein-protein interaction network for variation in alcohol sensitivity. The network was constructed from candidate genes (red ovals), identified from the combined DGRP and extreme QTL GWA analyses, while allowing for one missing gene (grey ovals). The candidate genes with the most interactions are depicted on the perimeter of the interaction network. (PDF 825 kb) Additional file 12:
**Quantitative qRT-PCR primer sequences.** (XLSX 11 kb)

## Abbreviations

AIP: Advanced Intercross Population
DGRP: *Drosophila melanogaster* Genetic Reference Panel
MET: Mean elution time
E1: Acute exposure to alcohol
E2: Repeated exposure to alcohol
T: Tolerance
GWA: Genome-wide association
SNP: Single nucleotide polymorphism
