# Supplementary material for: Polymorphisms in early neurodevelopmental genes affect natural variation in alcohol sensitivity in adult drosophila
Source: BMC Genomics. 2015 Oct 26;16:865. doi: 10.1186/s12864-015-2064-5 (PMC4624176; doi:10.1186/s12864-015-2064-5)
Supplement: Additional file 1: Table S1. — Analyses of variance of alcohol sensitivity of 205 DGRP lines. (DOCX 20 kb) [file 12864_2015_2064_MOESM1_ESM.docx]

**Table S1. Analyses of variance of alcohol sensitivity of 205 DGRP lines.**

| **Analysis** | **Source** | **DF** | **MS** | **F** | ***P*-Value** | **Variance Component** | ***H*^2^** | ***r_G_*** |  |
| --- | --- | --- | --- | --- | --- | --- | --- | --- | --- |
| **Exposure 1**  **(Sexes Pooled)** | *L* | 204 | 1413.14 | 8.24 | <0.0001 | *σ_L_*^2^ = 4.0 | 0.42 | *r_GS_* = 0.83 |  |
|  | *S* | 1 | 8602.24 | 51.36 | <0.0001 | Fixed |  |  |  |
|  | *L*×*S* | 204 | 171.07 | 3.91 | <0.0001 | *σ_LS_*^2^ = 0.82 |  |  |  |
|  | *Rep(LxS)* | 484 | 44.15 | 6.59 | <0.0001 | *σ_R(LS)_*^2^ = 0.5 |  |  |  |
|  | *Error* | 67100 | 6.7 |  |  | *σ_E_*^2^ = 6.7 |  |  |  |
| **Exposure 2**  **(Sexes Pooled)** | *L* | 204 | 1622.65 | 8.41 | <0.0001 | *σ_L_*^2^ = 5.1 | 0.38 | *r_GS_* = 0.83 |  |
|  | *S* | 1 | 3993.57 | 21.28 | <0.0001 | Fixed |  |  |  |
|  | *L*×*S* | 204 | 192.69 | 3.94 | <0.0001 | *σ_LS_*^2^ = 1.03 |  |  |  |
|  | *Rep(LxS)* | 475 | 49.41 | 4.94 | <0.0001 | *σ_R(LS)_*^2^ = 0.61 |  |  |  |
|  | *Error* | 58454 | 10.01 |  |  | *σ_E_*^2^ = 10.01 |  |  |  |
| **Females (Exposures Pooled)** | *L* | 204 | 1450.06 | 5.63 | <0.0001 | *σ_L_*^2^ = 3.99 | 0.38 | *r_GE_* = 0.76 |  |
|  | *E* | 1 | 135251.98 | 539.74 | <0.0001 | Fixed |  |  |  |
|  | *L*×*E* | 204 | 257.44 | 4.85 | <0.0001 | *σ_LE_*^2^ = 1.26 |  |  |  |
|  | *Rep(LxE)* | 482 | 53.36 | 6.68 | <0.0001 | *σ_R(LE)_*^2^ = 0.64 |  |  |  |
|  | *Error* | 65000 | 7.99 |  |  | *σ_E_*^2^ = 7.99 |  |  |  |
| **Males (Exposures Pooled)** | *L* | 204 | 1447.04 | 5.43 | <0.0001 | *σ_L_*^2^ = 4.18 | 0.39 | *r_GE_* = 0.73 |  |
|  | *E* | 1 | 108672.60 | 423.04 | <0.0001 | Fixed |  |  |  |
|  | *L*×*E* | 204 | 266.44 | 6.71 | <0.0001 | *σ_LE_*^2^ = 1.54 |  |  |  |
|  | *Rep(LxE)* | 477 | 40.08 | 4.71 | <0.0001 | *σ_R(LE)_*^2^ = 0.47 |  |  |  |
|  | *Error* | 60554 | 8.51 |  |  | *σ_E_*^2^ = 8.51 |  |  |  |
| **Scaled Tolerance**  **Sexes Pooled)** | *L* | 204 | 1.52 | 4.88 | <0.0001 | *σ_L_*^2^ = 0.29 | 0.76 | *r_GS_* = 0.76 |  |
|  | *S* | 1 | 0.08 | 0.28 | 0.6 | Fixed |  |  |  |
|  | *L*×*S* | 204 | 0.31 | 2.84 | <0.0001 | *σ_LS_*^2^ = 0.09 |  |  |  |
|  | *Error* | 471 | 0.11 |  |  | *σ_E_*^2^ = 0.11 |  |  |  |

*L*: DGRP line. *S*: Sex. *Rep*: Replicate. *E*: Exposure. DF: degrees of freedom. MS: Mean Squares. F: F-statistic. *H*^2^: broad sense heritability. *r_G_*: genetic correlation (*r_GS_* = cross sex genetic correlation; *r_GE_* = cross-environment genetic correlation).
