# Supplementary figures and images for: Polymorphisms in early neurodevelopmental genes affect natural variation in alcohol sensitivity in adult drosophila
Source: BMC Genomics. 2015 Oct 26;16:865. doi: 10.1186/s12864-015-2064-5 (PMC4624176; doi:10.1186/s12864-015-2064-5)

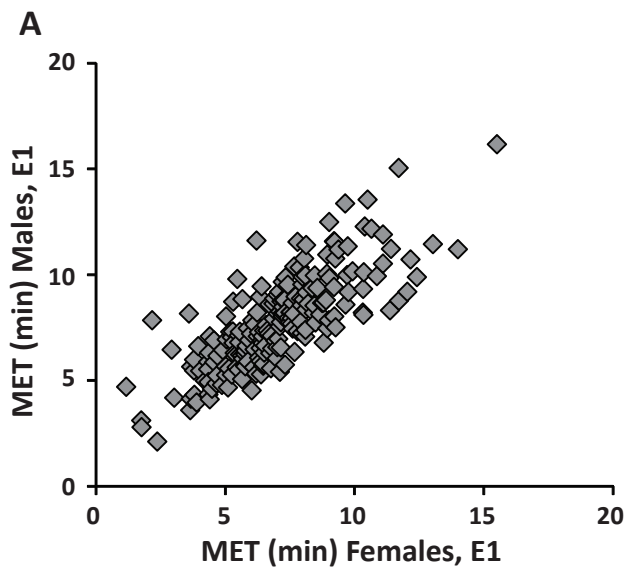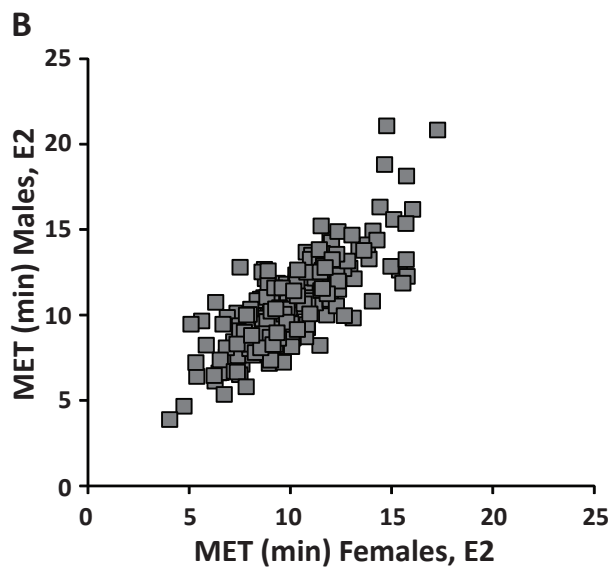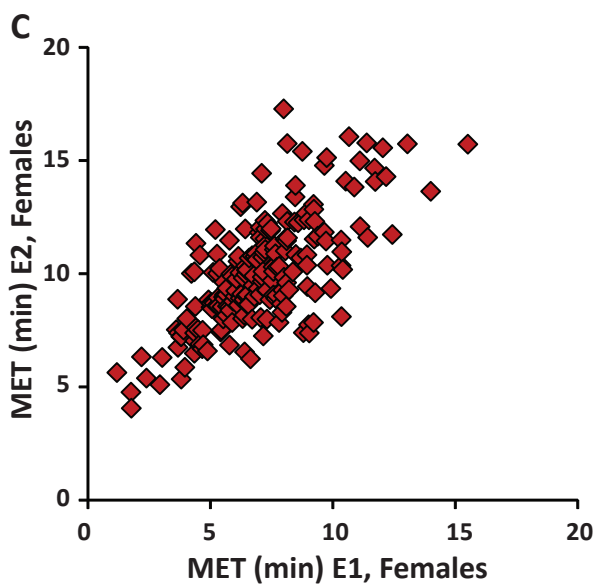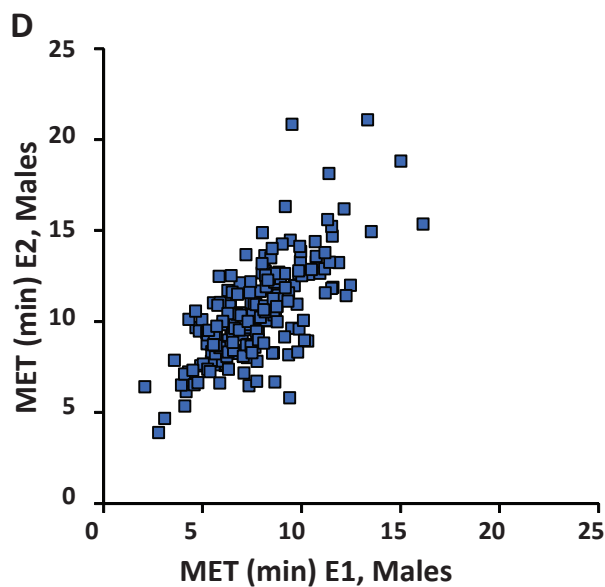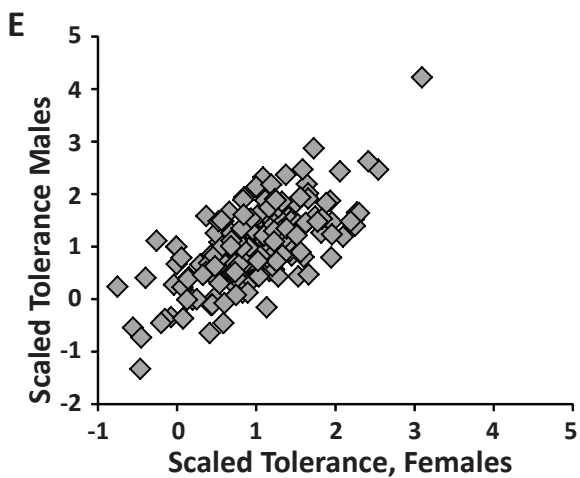

Supplement: Additional file 3: Figure S1. — Phenotypic correlations among METs in males and females and across treatments. (A) E1 females and males (r P = 0.78). (B) E2 females and males (r P = 0.79). (C) Females, E1 and E2 (r P = 0.7). D) Males, E1 and E2 (r P = 0.7). (E) Tolerance, females and males (r P = 0.67). (PDF 313 kb) [file 12864_2015_2064_MOESM3_ESM.pdf]

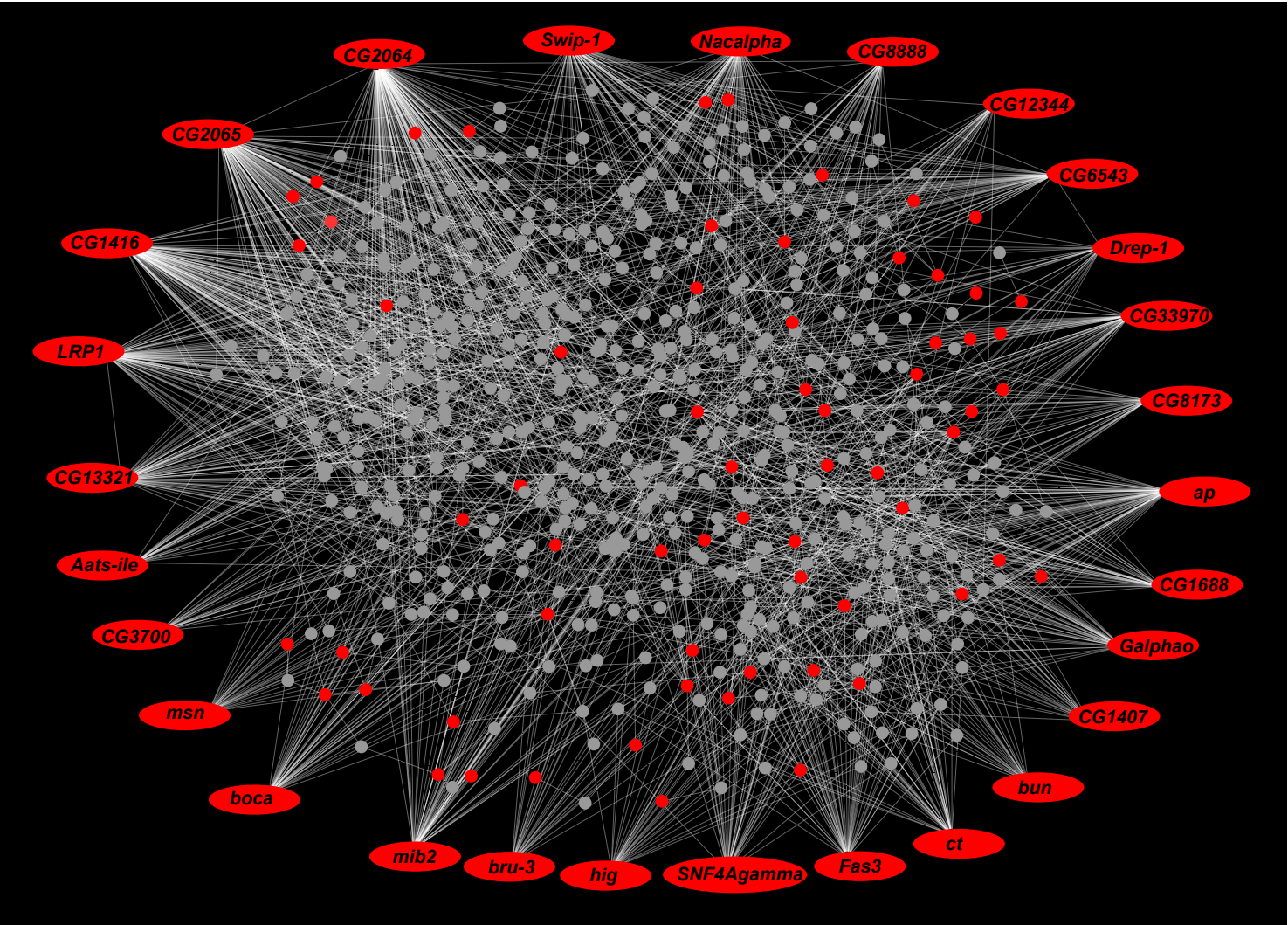

Supplement: Additional file 11: Figure S2. — Protein-protein interaction network for variation in alcohol sensitivity. The network was constructed from candidate genes (red ovals), identified from the combined DGRP and extreme QTL GWA analyses, while allowing for one missing gene (grey ovals). The candidate genes with the most interactions are depicted on the perimeter of the interaction network. (PDF 825 kb) [file 12864_2015_2064_MOESM11_ESM.pdf]
